# adLIMS: a customized open source software that allows bridging clinical and basic molecular research studies

**DOI:** 10.1186/1471-2105-16-S9-S5

**Published:** 2015-06-01

**Authors:** Andrea Calabria, Giulio Spinozzi, Fabrizio Benedicenti, Erika Tenderini, Eugenio Montini

**Affiliations:** 1 San Raffaele Scientific Institute, Division of Regenerative Medicine, Stem Cells, and Gene Therapy, HSR-TIGET, The San Raffaele Telethon Institute for Gene Therapy, Milan, Italy; 2 Department of Informatics, Systems and Communication (DISCo), University of Milano-Bicocca (UNIMIB), Milan, Italy

**Keywords:** LIMS, Open Source Software, Information Systems, ADempiere ERP, Sample Tracking

## Abstract

**Background:**

Many biological laboratories that deal with genomic samples are facing the problem of sample tracking, both for pure laboratory management and for efficiency. Our laboratory exploits PCR techniques and Next Generation Sequencing (NGS) methods to perform high-throughput integration site monitoring in different clinical trials and scientific projects. Because of the huge amount of samples that we process every year, which result in hundreds of millions of sequencing reads, we need to standardize data management and tracking systems, building up a scalable and flexible structure with web-based interfaces, which are usually called Laboratory Information Management System (LIMS).

**Methods:**

We started collecting end-users' requirements, composed of desired functionalities of the system and Graphical User Interfaces (GUI), and then we evaluated available tools that could address our requirements, spanning from pure LIMS to Content Management Systems (CMS) up to enterprise information systems. Our analysis identified ADempiere ERP, an open source Enterprise Resource Planning written in Java J2EE, as the best software that also natively implements some highly desirable technological advances, such as the high usability and modularity that grants high use-case flexibility and software scalability for custom solutions.

**Results:**

We extended and customized ADempiere ERP to fulfil LIMS requirements and we developed *adLIMS*. It has been validated by our end-users verifying functionalities and GUIs through test cases for PCRs samples and pre-sequencing data and it is currently in use in our laboratories. *adLIMS *implements authorization and authentication policies, allowing multiple users management and roles definition that enables specific permissions, operations and data views to each user. For example, *adLIMS *allows creating sample sheets from stored data using available exporting operations. This simplicity and process standardization may avoid manual errors and information backtracking, features that are not granted using track recording on files or spreadsheets.

**Conclusions:**

*adLIMS *aims to combine sample tracking and data reporting features with higher accessibility and usability of GUIs, thus allowing time to be saved on doing repetitive laboratory tasks, and reducing errors with respect to manual data collection methods. Moreover, *adLIMS *implements automated data entry, exploiting sample data multiplexing and parallel/transactional processing. *adLIMS *is natively extensible to cope with laboratory automation through platform-dependent API interfaces, and could be extended to genomic facilities due to the ERP functionalities.

## Background

In many biological laboratories, sample tracking is an outstanding issue and often represents a bottleneck for the correct handling and interpretation of experimental data. This issue is becoming particularly critical when automation and high-throughput technologies are introduced in the laboratory practice. Our laboratory performs high-throughput characterization of vector-genomic integration sites in the context of gene therapy applications based on the delivery of therapeutic genes by viral vectors that stably integrate into the genome of targeted cells, as well as gene therapy preclinical models and insertional mutagenesis research projects [1-9]. Vector integration sites are retrieved and mapped in the genome through a combination of Polymerase Chain Reaction (PCR)-based techniques [10], next generation sequencing (NGS) and bioinformatics analyses [11]. We process and analyze around 2000 samples/year resulting in hundreds of millions of sequencing reads. Despite the fact that adopting robotic automation for sample manipulation in our laboratory has provided many advantages in terms of manual error-reduction and data production scalability, drawbacks related to sample information volume and tracking are still present. These reasons prompted us to develop a Laboratory Information Management System (LIMS) [12] for sample tracking on a scalable and flexible infrastructure with an easily accessible and web-based interface. LIMS is a type of information system implemented as a software utility specifically designed to improve the data acquisition and sample monitoring along laboratory workflows, and supporting sample reporting. An information system is a combination of information technologies developed to grant business processes efficiency and monitoring. Extended IS are the Enterprise Resource Planning (ERP) solutions [13] that integrate the standard information system features with accounting and administrative operations for performance monitoring through dashboards and data mining tools.

In this work we describe our LIMS, developed on an existing open source ERP framework that natively implements all technological functionalities, as software customization and parameterization. After a brief introduction of the ERP framework with the motivation of the specific choice, we will describe in details our implementation with custom use cases and scenarios derived from our laboratory requirements and experience.

## Methods

We followed a typical software engineering approach, "waterfall" [14], to design our solution. We first collected end-users' requirements, composed of desired functionalities of the systems and graphical user interfaces (GUI). Then we evaluated available tools that could address our requirements, spanning from pure LIMS to Content Management Systems (CMS) up to enterprise information systems. In the next step we selected our candidate solution and we designed its configuration to best fulfil our requirements.

### Requirements Analysis

The analysis of requirements, also called requirements engineering, is the process of acquiring software expectations from users/clients in terms of functionalities and interaction that are translated in software requirements [15].

**Figure 1:**
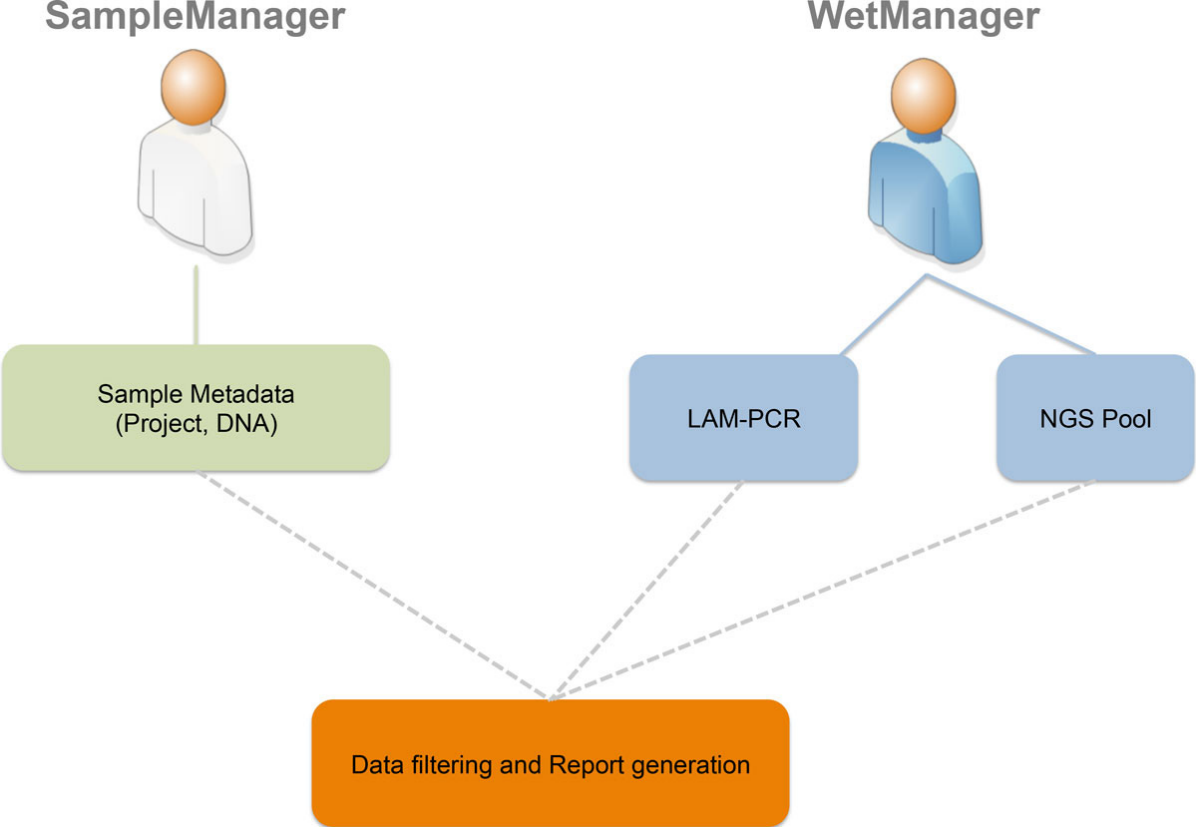
****adLIMS' use case **The UML Use Case Diagram of our case study**. In our case we have two actors: ***SampleManager ***and ***WetManager***. A user with ***SampleManager ***role can insert, edit, manage, search and filter only clinical data, and deals with data about DNA, project and patient. A user with ***WetManager ***role can view clinical data, and it can insert, edit, manage, search and filter data about LAM-PCR and NGS pool.

The first step is to define which types of users will access the LIMS (roles and groups) and which authorization policies we have to apply to specific users (permissions on data and operations). Using a Unified Modeling Language (UML) [16] use case diagram (Figure 1), we collected desired roles (called "actors" in UML) and operations (called "actions" in UML). The two principal roles of the system are distinguished for the area in which they operate. In this instance we identified two main areas: a sample management area where samples are collected from patients or subjects and a wet experimental area in which collected samples are processed to retrieve vector-cellular genomic junctions. For this reason we identified two corresponding roles, a *SampleManager *and a *WetManager*, and their permissions and operations scope correspond to the data that they can access: the *SampleManager *role is related to users that input and manage sample metadata (project, DNA and so on), while the *WetManager *role is related to users that input and manage experimental procedures and workflows on the DNA samples (PCR steps, NGS pooling, and so on). Then we collected the desired authorizations associated to each role as reported in the CRUD (Create, Read, Update, Delete) policies (Table 1).

**Table 1:** **User's Rules in "Create, Read, Update and Delete" format (CRUD)**. The table is a summary of the role permissions and policies in ***adLIMS ***in terms of data access. We use the CRUD syntax: create (C), read (R), update (U), delete (D).

	Clinic data	Sequencing data	Projects	LIMS managing	Users managing
*WetManager*	R	CRUD	CRUD	--	--

*SampleManager*	CRUD	--	CRUD	--	--

*Administrator*	--	--	--	CRUD	CRUD

The *SampleManager *role can access and edit metadata related to all clinical and preclinical aspects such as anonymized patients' IDs, DNA (concentrations, volume, and so on) and cell types (markers, lineage, and so on). The visibility spectrum of a user with the *SampleManager *role is restricted to sample metadata only and will not be able to read other information of post sample processing.

The *WetManager *role is able to control all aspects related to experimental procedures and workflows on the samples. A user assigned to the *WetManager *role will not be able to modify input metadata from the *SampleManager *role.

We added an *administrator *role (or *SuperUser*) that is able to control and edit every component of the system, customizing all the functional and graphical levels related to the model-view-controller paradigm [17]. However this role is not authorized to modify input data. At the functional level, the *administrator *has the privileges to: (1) create, delete, and suspend accounts and (2) define rules to access to the database (such as tables, views, fields, etc.). At the graphical level, the *administrator *can create and edit windows and layouts, control how to graphically visualize and access data and define how to export and import data in the information system.

**Figure 2:**
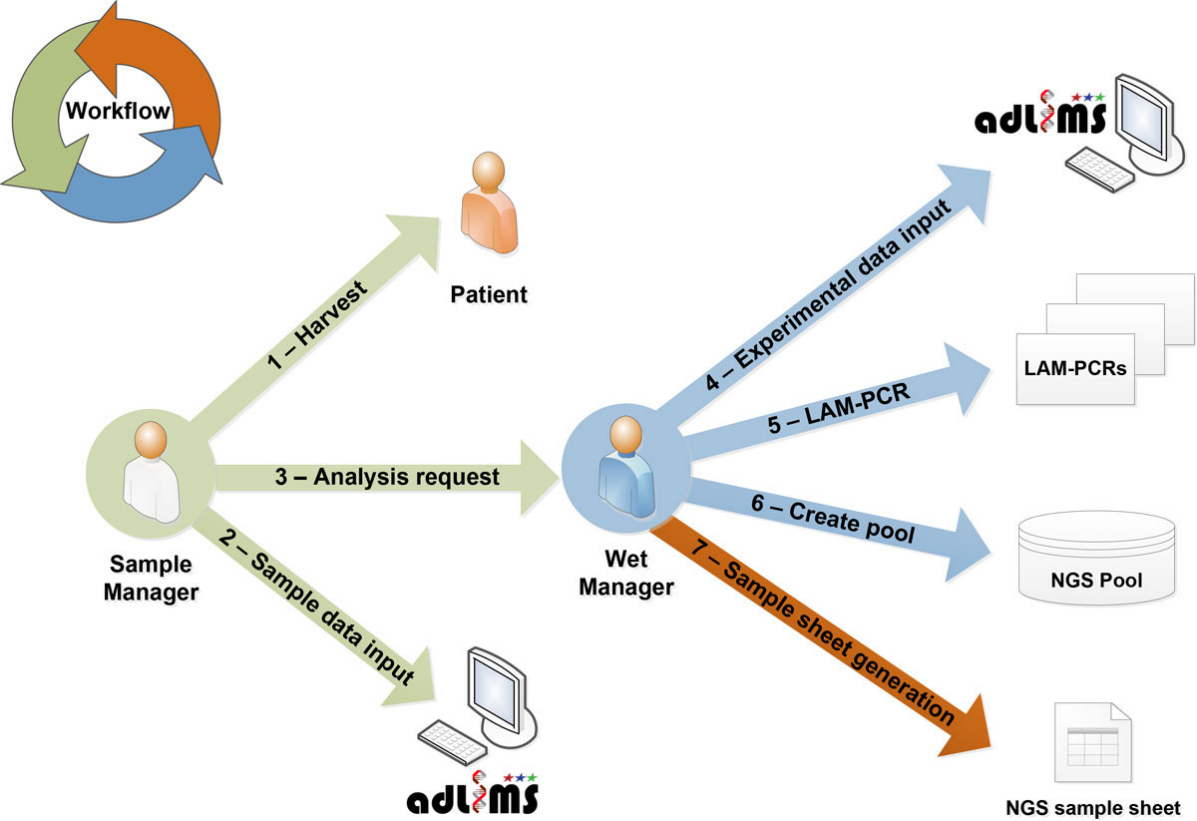
****Sample case study of *adLIMS ***A user with ***SampleManager ***role inserts data of the sample harvested from a patient (in this scenario) into ***adLIMS ***and then activate a request for wet processing (arrow number 3)**. A user with ***WetManager ***role processes the sample first adding other sample related information like data quality or concentration, then sets-up a LAM-PCR experiment (arrow number 5), prepares a pool for sequencing (arrow number 6), and finally exports a sample sheet. The arrow colors represent different conceptual steps of the workflow.

From this analysis of requirements we derived the interaction workflow (Figure 2). In the first step of our workflow, a user with a *SampleManager *role inputs data related to the specific project and the samples that need to be processed for integration site analysis, in particular information relative to DNA. These samples will be then processed (upon a request of analysis from the *SampleManager*) in the custom workflow by a user with a *WetManager *role for linear amplification mediated (LAM)-PCR [10]. The procedure of the LAM-PCR protocol has to be implemented in a LIMS process that allows tracking each sample manipulation. LAM-PCR products are then selected and pooled for deep sequencing. All the information of the pooled products are exported and formatted as required by Illumina MiSeq sample sheet. Therefore the information system allows monitoring and tracking sample metadata, LAM-PCR processing and the pool preparation for NGS supporting DNA barcodes association without directly handling NGS files. Additional file 1 summarizes the laboratory workflow and system interaction based on the above-mentioned requirements.

As additional requirements, the system has to be web-based and supported by reliable technologies with a backup system thus enabling data maintenance and recovery in case of failures (e.g. electrical supply problems). The centralized nature of the system requires standard hardware performances such as the simultaneous interaction with dozens of users keeping response times faster than 3 seconds.

### Evaluation of available LIMS and alternative solutions

We evaluated available LIMS solutions, from commercial to open source ones. Commercial or stand-alone LIMSs [18,19] are often very expensive and/or lack the flexibility and scalability needed to manage different types of sample data, procedures and analyses specifically designed for each research project. We also explored open source software designed for biological laboratories, such as Bika LIMS [20], LabKey [21] or Galaxy [22], and content management systems (CMS) like Plone [23] and Drupal [24] under the perspective of customizing them and exploiting built-in functionalities such as user management, workflow management and configuration. Unfortunately, none of them fully satisfied our requirements because most of the features, that are the peculiarity of a LIMS (like export, report, etc.), have not been implemented. In this context, we analyzed Enterprise Resource Planning (ERP) solutions and ADempiere ERP [25] was the best available software. ADempiere ERP has been developed under GPL license in Java J2EE with Model-View-Controller design pattern [17] and database-driven logic [26]. ADempiere ERP implements all required features and presents some highly desirable technological advances (see Additional file 2 for the list of desirable features with a comparative analysis among Bika LIMS, LabKey and ADempiere), such as high usability (web and mobile interfaces) and modularity that grants high use-case flexibility (plug and play approach) and software scalability for custom solutions (adaptable to all use cases). The application server is JBoss and supported databases are Oracle and PostgreSQL. Web interfaces exploit the latest technologies with ZK [27]. Moreover, it natively supports multiple languages, accounting procedures and dashboards for process monitoring thanks to the ERP functionalities that can be easily adapted to high-standard industrial and commercial contexts.

### Design

The last process in the "waterfall" approach is the system design. In order to translate the requirements into both functionalities and GUI within ADempiere ERP, we developed an extension of the core database by applying the required operations and user's policies resulting in a scalable system that natively supports Java fat client and web interfaces. For each end-user interaction and functionality, we designed a custom view of the workflow (Figure 2) with dedicated GUIs based on use cases (Figure 1). Since ADempiere ERP is database-driven, the design of the database related to the LIMS extension is a key aspect that drives and manages both the workflow instance and the GUIs. Our database extension is compliant with the core-system table design that required the addition of ten pre-defined fields (see Additional file 3). This operation is required because ADempiere ERP leverages on inner tables to directly create forms (windows) and GUIs. The "application dictionary" is one of the most powerful aspects of ADempiere ERP that acts as the engine of the database-driven model. All metadata needed to build data forms, windows and GUI are contained in the application dictionary that operates at the application layer and generates windows, tabs, menu, forms, nested elements connection, and so on. The application dictionary allows dynamic and flexible changes in GUI and data forms by changing its table content without requiring programming development that is thus drastically reduced. As a direct consequence, GUI changes or customizations can be configured directly in the application dictionary without requiring software compiling or re-building. For example, to create a new window with proper title, menu bar, tool bar, and status bar, ADempiere ERP automatically adds elements in the application dictionary and generates all required fields starting from the database table.

Based on the general workflow modeled in the analysis of requirements (Figure 2), we designed and implemented the LIMS database as an extension of the core ADempiere ERP database (Additional file 4), here reported as Entity-Relationship model in which each basic entity is associated to a custom table ("project", "subject", "DNA", "vector", "sample", and so on). We implemented the model in PostgreSQL and we used *BLOB *(Binary Large Object) to manage external files as attachments (such as images, pdf files, and so on) that users can upload into any entry. We then created custom GUIs related to the previously described workflow for the management of all data tables (Figure 2). Each window and the relative data are accessed by users according to their role (*SampleManager*, *WetManager *and *administrator *role) and authorization policies. Is always possible to modify existing roles and names and to add new roles according to new requirements or specifications. In our laboratory practice we routinely collect LAM-PCR data, gel images and sequencing quality reports and we store these data in *adLIMS*. The LAM-PCR workflow has been automated by implementing dedicated tables in the system ("experiment", "lam_pcr_linear", "lam_pcr_1st_exp", "lam_pcr_2nd_exp") with corresponding GUI input forms. Custom database triggers support the multiplexing of samples during the experimental operations required in different steps of the process (see Additional file 3 for trigger details). Similar automated procedures have been developed to support the generation of sequencing pools for high-throughput NGS platforms by defining custom tables ("fusion", "pool", "pool_details") and associated GUI. To avoid hard drive bottlenecks, raw NGS files are included in the system as links (absolute path with host server) and not as attachments.

## Results and Discussion

**Figure 3:**
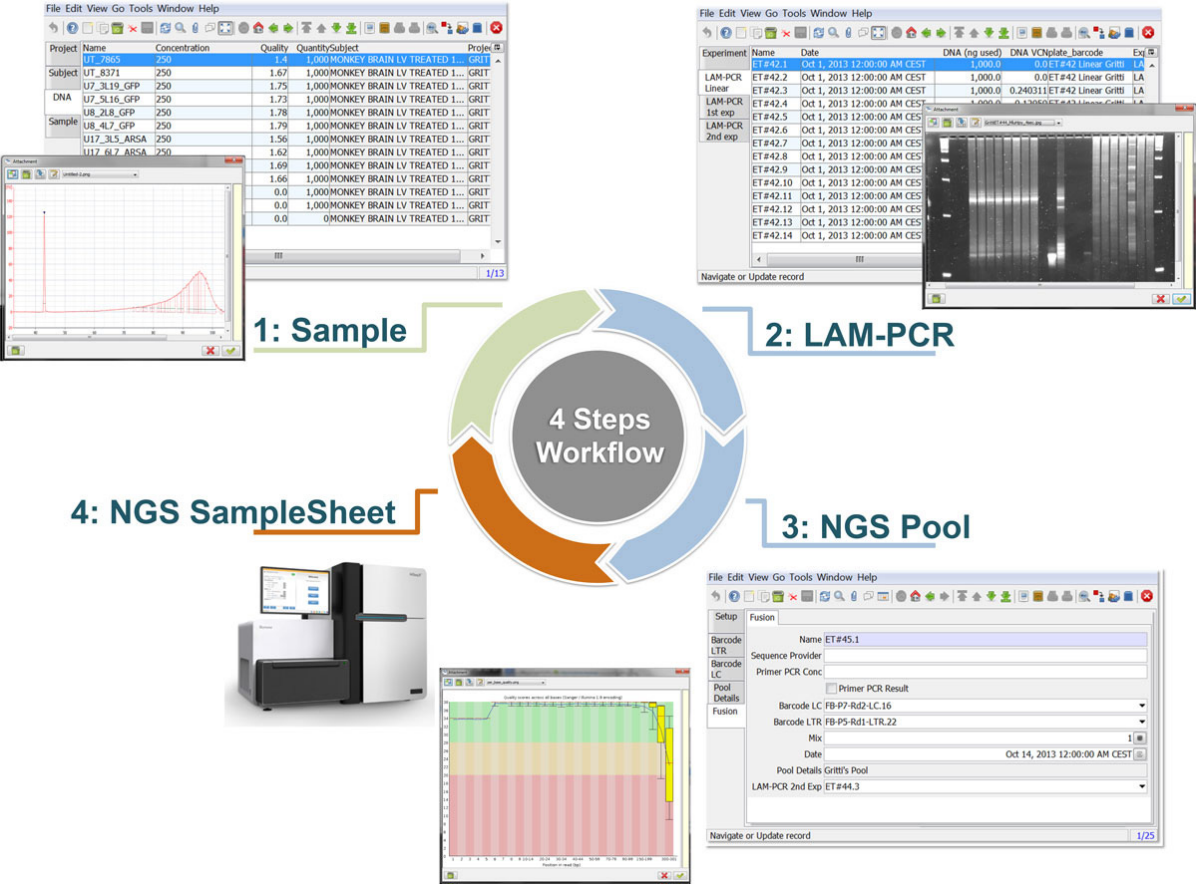
***adLIMS *workflow**. The circular schema represents the ***adLIMS ***workflow from sample input to NGS pool generation. In the first step, a user with the ***SampleManager ***role fills the "Cell" and "Sample" forms that contain data about tissue source, lineage, cell type, cell marker, project, subject, DNA metadata. The two subplots related to this step highlight examples of real input data, in which on the back is visible a sample DNA list in the context of the ***adLIMS ***window layout whereas on the foreground is opened a sample image of a DNA quantification from an input data, stored as attachment in ***adLIMS***. In the second step a user with the ***WetManager ***role processes samples along the LAM-PCR protocol setting-up the required Linear, 1° and 2° Exponential PCR amplification. Here the two subplots represent a sample window layout on the back and a picture of an agarose gel electrophoresis of LAM-PCR products in foreground. In the third step a subset of PCR samples are pooled together through the NGS Pool Setup and the sample window layout allows visualizing all required fields of the forms. The final step is the NGS sequencing and sample sheet acquisition, in which is possible to upload raw data quality reports such as the per-base sequence quality visible on the windowed subplot.

We developed *adLIMS *as an open source customization of ADempiere ERP, which implements the general workflow and requirements previously described (Figure 3 and Additional file 5 for details of roles and GUIs). *adLIMS *exploits all the core functionalities of ADempiere resulting in a completely database-driven solution that, from the extended database, is able to build forms and GUIs. We maximized the use of itemized form elements (dropdown menus, checkboxes, etc.) as GUI's design principle to reduce users' errors or typos and we limited the free text boxes as much as possible applying some basic form validators (e.g. e-mail check). As a direct consequence of the authorization policies, we restricted the user interaction and scope to the specific sections they are supposed to use with dedicated privileges. Each user can access *adLIMS *both using web GUI and the Java fat client. The web client is the most used interface that does not require any installation on the target user machine nor requires any configuration and all computational requests are processed server-side lightening client-side workload. Moreover *adLIMS *allows mobile interfaces for smartphones and tablets, features inherited from ADempiere ERP. We implemented data backup by creating custom server side jobs, scheduled as incremental backup with daily dump of the whole database.

**Figure 4:**
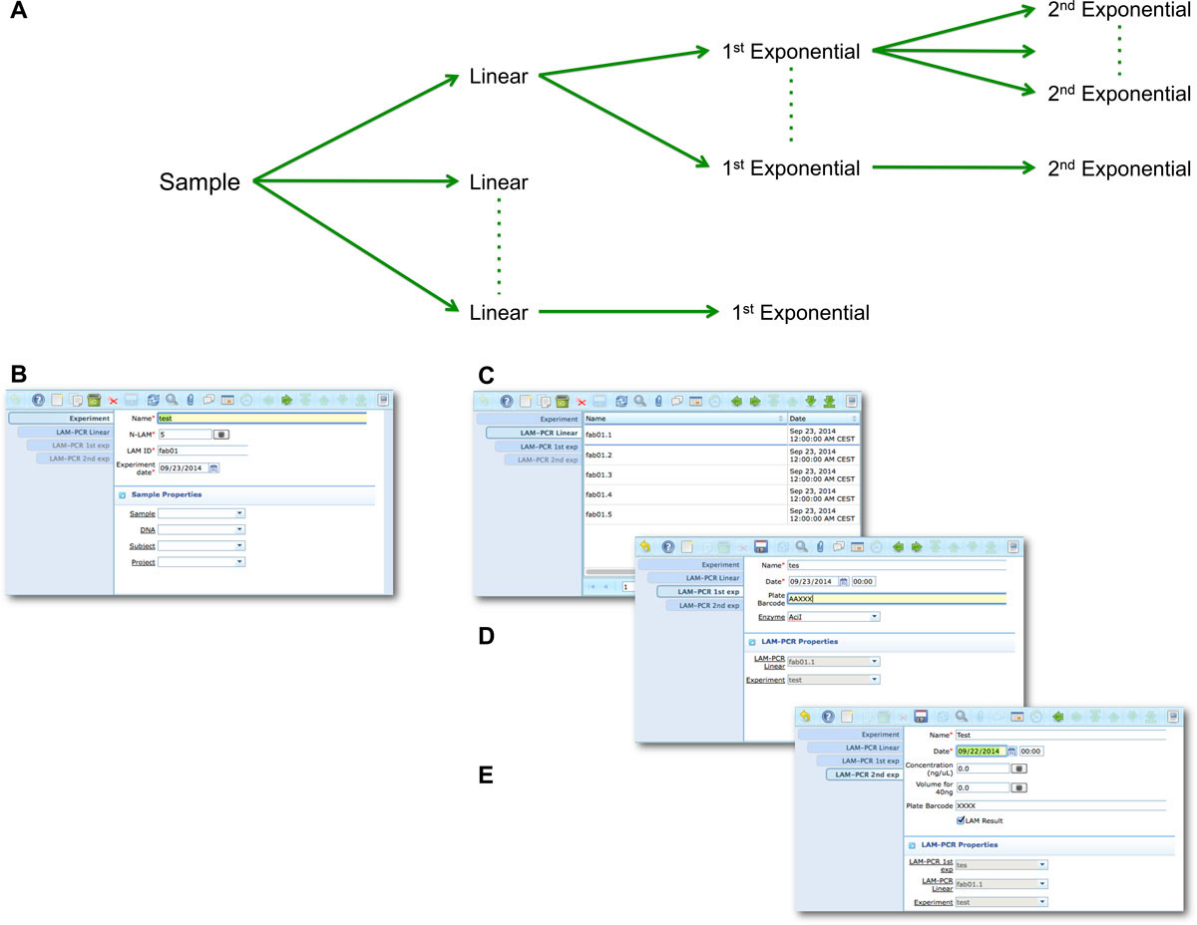
**LAM-PCR workflow and the corresponding *adLIMS *automated process**. The LAM-PCR procedure [10] is reported as diagram (A) in which from a selected sample, different linear amplifications may be processed followed then by a first and a second exponential PCR amplification. The procedure is developed in ***adLIMS ***starting from the experiment set-up (B) in which a user fills the form with the data relative to the experiment such as "Name", number of LAM-PCRs (N-LAM), "LAM ID" and "date". Then ***adLIMS ***creates a number of entries as reported in the field "N-LAM", for the subsequent step "LAM-PCR linear" (C), that will be then processed and expanded in the "LAM-PCR 1**^st ^**exp" step (D) with details such as "Name", "Plate barcode" and "Enzyme". The last step is the "LAM-PCR 2**^nd ^**exp" (E), corresponding to the second exponential amplification, in which the user completes additional data of the experiment ("concentration" and "quality").

The LAM-PCR and pre-sequencing activities are directly supported by the *adLIMS *automated procedures that allow the creation of parallel processes by database triggers, required in case of laboratories with high multiplexing needs (such as PCRs liquid handler with 96 wells/plate format). For the LAM-PCR workflow (Figure 4A), *adLIMS *supports the user with dedicated GUI (Figure 4B).

**Figure 5:**
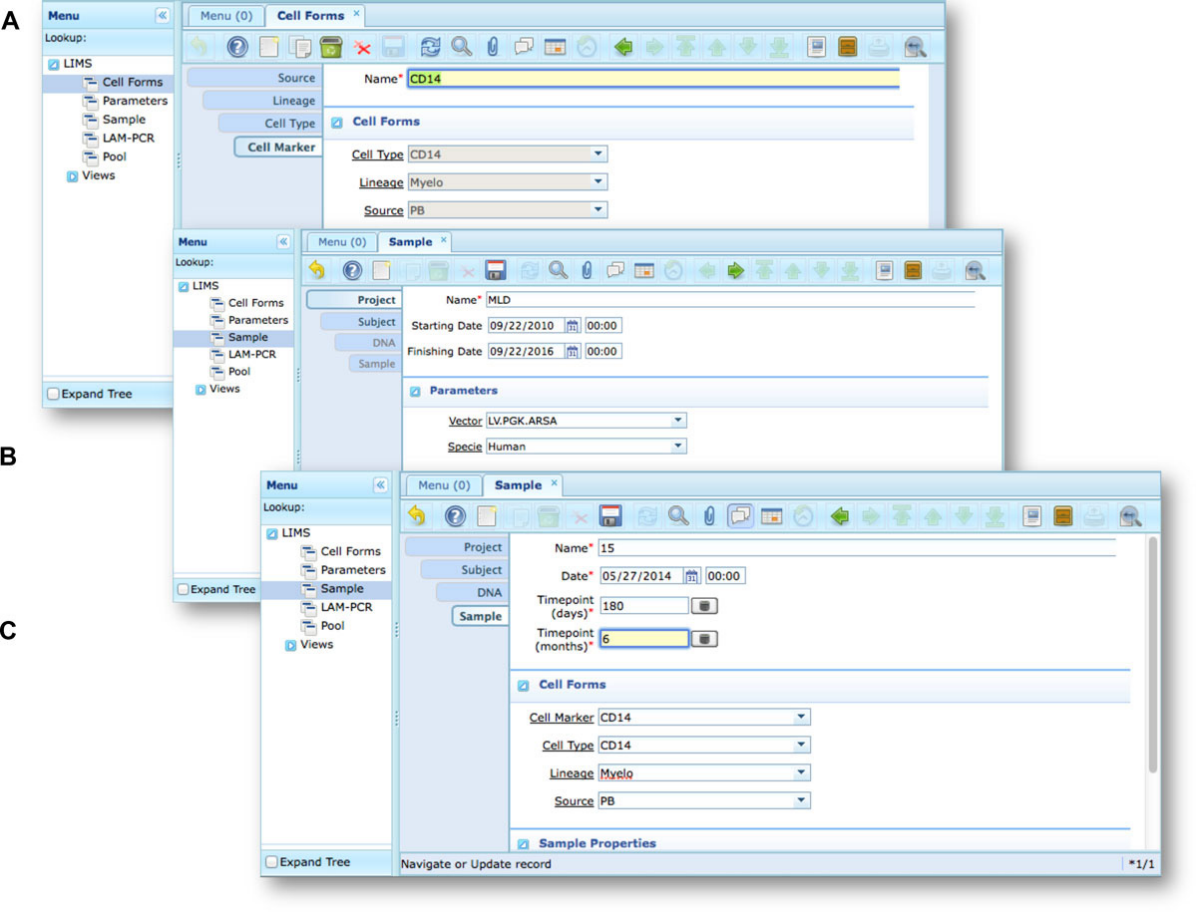
****Workflow scenario of a user with *SampleManager *role **An example of an ***adLIMS' ***workflow for the ***SampleManager ***role in the context of the web interface in ZK**. (A) The Cell Form window is filled with some details of the Cell Marker tab, reached from the previous related tabs (Source, Lineage, Cell Type) after user log-in. (B) The Sample window filled with some details of a project in which an example of specie dropdown menu is expanded (data from the related table). (C) The Sample window filled with some details of the tab Sample.

**Figure 6:**
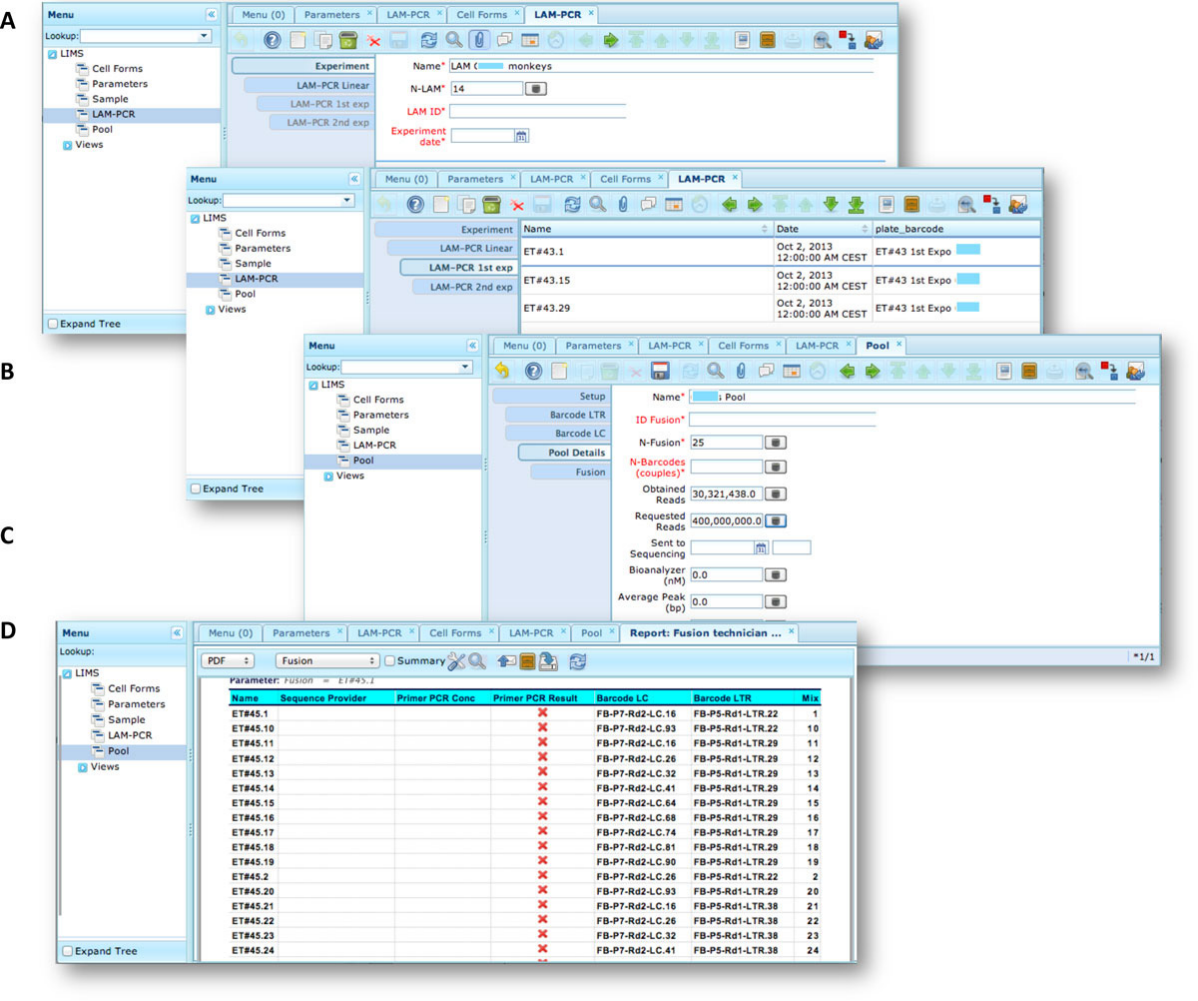
****Workflow scenario of a user with *WetManager *role **An example of an ***adLIMS' ***workflow for the ***WetManager ***role in the context of the web interface in ZK where a user with the ***WetManager ***role can insert/edit/delete data about the LAM-PCR process and obtain the report of the selected LAM_PCR experiments (pool) to sequence**. (A) The LAM-PCR window filled with some details of a new experiment tab after user log-in. (B) The LAM-PCR window filled with some details of a first exponential data reached from the previous related tabs (Experiment and Linear). (C) The Pool window filled with some details of the tab Pool Details reached from the previous related tabs (Setup, Barcode LTR, Barcode LC). (D) Example of exported report in PDF file format from samples of a pool.

Our end-users validated *adLIMS *by verifying functionalities and GUIs through test cases for PCRs samples and pre-sequencing data. Here we reported two settings related to two distinct sections of the general workflow (Figure 3) with different scopes and actors, thus requiring different user policies and sample activities. The first workflow concerns the interaction of a user with *SampleManager *role that insert patient sample metadata into *adLIMS *(Figure 5). The system supports and guides the user to select and input the data with ad hoc forms, for example cell types (Cell Marker CD14, Myeloid in Figure 5A), tissues (peripheral blood, PB, in Figure 5A), DNA purity and concentration, project, sample ID and so on (Figure 5B-C). The *SampleManager *user profile is authorized to modify only the data relative to a specific project and subject from the menu list through two accessible windows ("Sample" and "Cell Forms"). In the second scenario, a user with the *WetManager *role is required to process DNA samples from specific cell populations (Figure 6), performing (1) LAM-PCR amplification (Figure 6A-B), (2) storing the images of the gel-electrophoresis PCR products, (3) preparing a sequencing pool (Figure 6C) and (4) exporting a report in PDF file format (Figure 6D). In all the steps of the process, *adLIMS *supports the user and enables the parallel sample processing and multiplexing.

**Figure 7:**
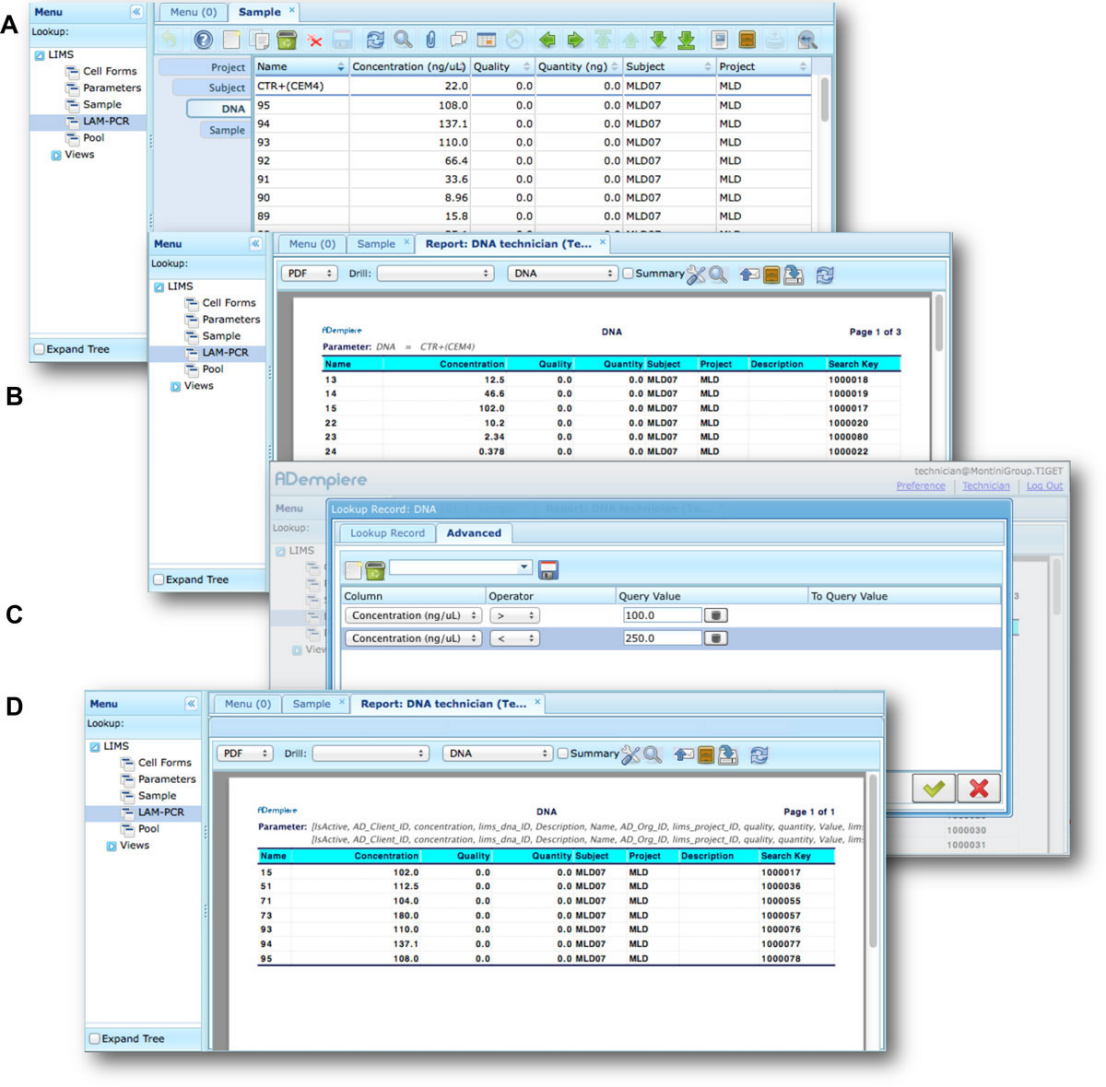
****Data filtering and reporting in *adLIMS ***The function of integrated reporting (via JasperReport) allows the users to configure customized reports from a selection of data fields**. In this example, a user starts from the DNA table (A), then clicks on the button "report" on the main menu to initiate the report generation, obtaining a new tab (B) with the list of the whole entries of the table. For example, to set a filter of the DNA samples with concentrations between 100 and 250 ng / uL, the user clicks on the "search" button (icon button with a magnifying glass) that opens a new window. Then the user switches in the advanced tab (C) of the search window and specifies the two needed filters. Once completed the search form, ***adLIMS ***filters the dataset and releases the PDF file that the user can save locally.

Moreover *adLIMS *allows report generation from all input data, both using all data and a selection of filtered data with user defined criteria (Figure 7). *adLIMS *exploits the data-driven logic to export data reports from tables to a wide range of file formats (PDF, XLS, CSV, etc.) through the JasperReports library [28]. JasperReports is a flexible report library that can be customized for each instance of the system. The default visualization allows reporting data in the same table view. In our daily practice, we use data reports to create sample-sheets of Illumina sequencing pools.

*adLIMS *in our laboratory practice has brought an improvement through simplification and traceability of the data entry in sample workflows and sample reporting with respect to traditional data storage methods. As an example, *adLIMS *allows to create a sample sheets by selecting samples information in a few clicks whereas without *adLIMS *this activity may require hours, potentially introducing typing errors.

*adLIMS *can be adapted and extended to any laboratory working with biological samples with limited efforts, which are mainly related to the database design and customization, thus defining the business and view logics (workflows and GUI) and creating the data structures (PostgreSQL or Oracle database schema and tables) that are automatically converted into interfaces and data views by the ADempiere ERP engine.

*adLIMS *currently does not provide direct interfaces with laboratory instruments but integrates local server connections (storage mount access, network printers, and so on). Future extensions of the system will include API integration of laboratory platforms for sequencing, PCR product quantification and liquid handlers. On the other hand, client side interfaces (such as barcode readers and scanners) can be directly integrated into *adLIMS*, enhancing sample tracking and data acquisition.

The potentialities of *adLIMS *in the context of genomic companies and facilities are conveyed in the ready-to-use availability of the ERP features that would only require customization.

## Conclusions

Currently, in many laboratories the procedures for data tracking and storage of sample information are based on spreadsheets without a real information management or standardization. This data management resulted in inefficiencies and redundancies, potentially generating many errors (typically typos) hard to backtrack or resolve. The use of a LIMS allows bypassing spreadsheets or local file management, supporting automation of all standard procedures for sample tracking. Exploiting *adLIMS*, our laboratory sustained the critical issues of sample tracking, data standardization and automation derived by the NGS and robotics revolution. This successful application of process engineering and monitoring allowed our laboratory to increase efficiency and reduce manual errors, thus posing concrete bases to sustain business scalability and potentially to approach pharmaco-vigilance monitoring of gene therapy patients in a highly standardized fashion compliant to regulatory requirements. Moreover *adLIMS *can natively be extended to incorporate ERP solutions, such as CRM, supply chain management, billing and accounting, integrated features that are critical for many genomics facilities.

## Competing interests

The authors declare that they have no competing interests.

## Authors' contributions

A.C. conceived of the study, participated in its design and coordination and drafted the manuscript. G.S. created the application (*adLIMS*) and drafted the manuscript. F.B., ET participated in the requirements analysis. E.T. tested the system. E.M. revised the manuscript and supervised the project. All authors read and approved the final manuscript.

## Availability and requirements

The source code, user guide and appliance of *adLIMS *are freely available at the project homepage http://sourceforge.net/projects/adlims. We also provide a live demo for users who want to evaluate *adLIMS *without installation. Release notes and other information will be also updated on the project homepage.

• **Project name**: adLIMS

• **Project homepage**: http://sourceforge.net/projects/adlims

• **Author**: Giulio Spinozzi

• **Operating system**: Platform independent

• **Supported web browsers**: Chrome, Firefox 3.5 +, Safari 4+

• **Programming language**: Java EE

• **Database**: PostgreSQL/Oracle

• **License**: GNU GPLv2

• **Any restrictions to use by non-academics**: None

## Supplementary Material

Additional file 1 **General data workflow of vector integration sites identification at TIGET Vector Integration Core **The standard flow of a vector integration site project in the Vector Integration Core at the Telethon Institute for Gene Therapy in Milan Click here for file

Additional file 2 **Features' comparison between Bika, LabKey and ADempiere **Comparison among the features available in Bika, LabKey and ADempiere. Click here for file

Additional file 3 **Customization set-up **The report file of all the customization steps required for creating an instance of ***adLIMS***, with an example of table creation and trigger definition. Click here for file

Additional file 4 ***adLIMS *entity-relationship database model *adLIMS ***database schema represented as Entity-Relationship model. Click here for file

Additional file 5 **Data elements managed by *adLIMS ***In this file we reported all data types inserted in adLIMS, following the workflow in Figure 3. Click here for file

## Declarations

Publication of this article has been funded by Telethon.

This article has been published as part of *BMC Bioinformatics *Volume 16 Supplement 9, 2015: Proceedings of the Italian Society of Bioinformatics (BITS): Annual Meeting 2014: Bioinformatics. The full contents of the supplement are available online at http://www.biomedcentral.com/bmcbioinformatics/supplements/16/S9.
