# Supplementary material for: adLIMS: a customized open source software that allows bridging clinical and basic molecular research studies
Source: BMC Bioinformatics. 2015 Jun 1;16(Suppl 9):S5. doi: 10.1186/1471-2105-16-S9-S5 (PMC4464029; doi:10.1186/1471-2105-16-S9-S5)

**adLIMS: a customized open source software that allows bridging clinical and basic molecular research studies**

Andrea Calabria^1^, Giulio Spinozzi ^1,2^, Fabrizio Benedicenti^1^, Erika Tenderini^1^, Eugenio Montini^1§^

^1^ San Raffaele Scientific Institute, Division of Regenerative medicine, Stem cells, and Gene therapy - HSR-TIGET - The San Raffaele Telethon Institute for Gene Therapy; Milan, Italy

^2^ Department of Informatics, Systems and Communication (DISCo) - University of Milano-Bicocca (UNIMIB); Milan, Italy

^§^Corresponding author

Email addresses:

AC: [calabria.andrea@hsr.it](mailto:calabria.andrea@hsr.it)

GS: [spinozzi.giulio@hsr.it](mailto:spinozzi.giulio@hsr.it)

FB: [benedicenti.fabrizio@hsr.it](mailto:benedicenti.fabrizio@hsr.it)

ET: [tenderini.erika@hsr.it](mailto:tenderini.erika@hsr.it)

EM: [montini.eugenio@hsr.it](mailto:montini.eugenio@hsr.it)

# Additional files

# Additional file 1

# General data workflow of vector integration sites identification at TIGET Vector Integration Core

In our laboratories, for each project of vector integration sites analysis, we first collect the metadata of the project and the DNA samples of the subjects (patients for clinical trials, mice for preclinical models, cell lines for insertional mutagenesis in vitro projects). This information is entered by a user with *SampleManager* role in the LIMS. Then a user with *WetManager* role uses the data previously entered to setup experiments of LAM-PCR and pre-sequencing. The data required for sequencing can be generated automatically from the LIMS which creates the sample sheet (useful for sequencing machines), and the association file (required for subsequent bioinformatics analysis of our pipeline [11]). The samples are then sequenced, typically with Illumina MiSeq platform, and data quality reports generated by FastQC are inserted in the LIMS into the corresponding entry of the pool. If the data quality of the sequencing pool is acceptable (threshold set to 15 in Phred scale), we run the analysis of the data through the pipeline that we developed [11] which identifies the vector-cellular genomic junctions (integration sites). The last step consists in statistical analyses project-dependent aimed at addressing target issues such as the monitoring safety and efficacy of gene therapy [2-3] or anti-cancer drug resistance [1].


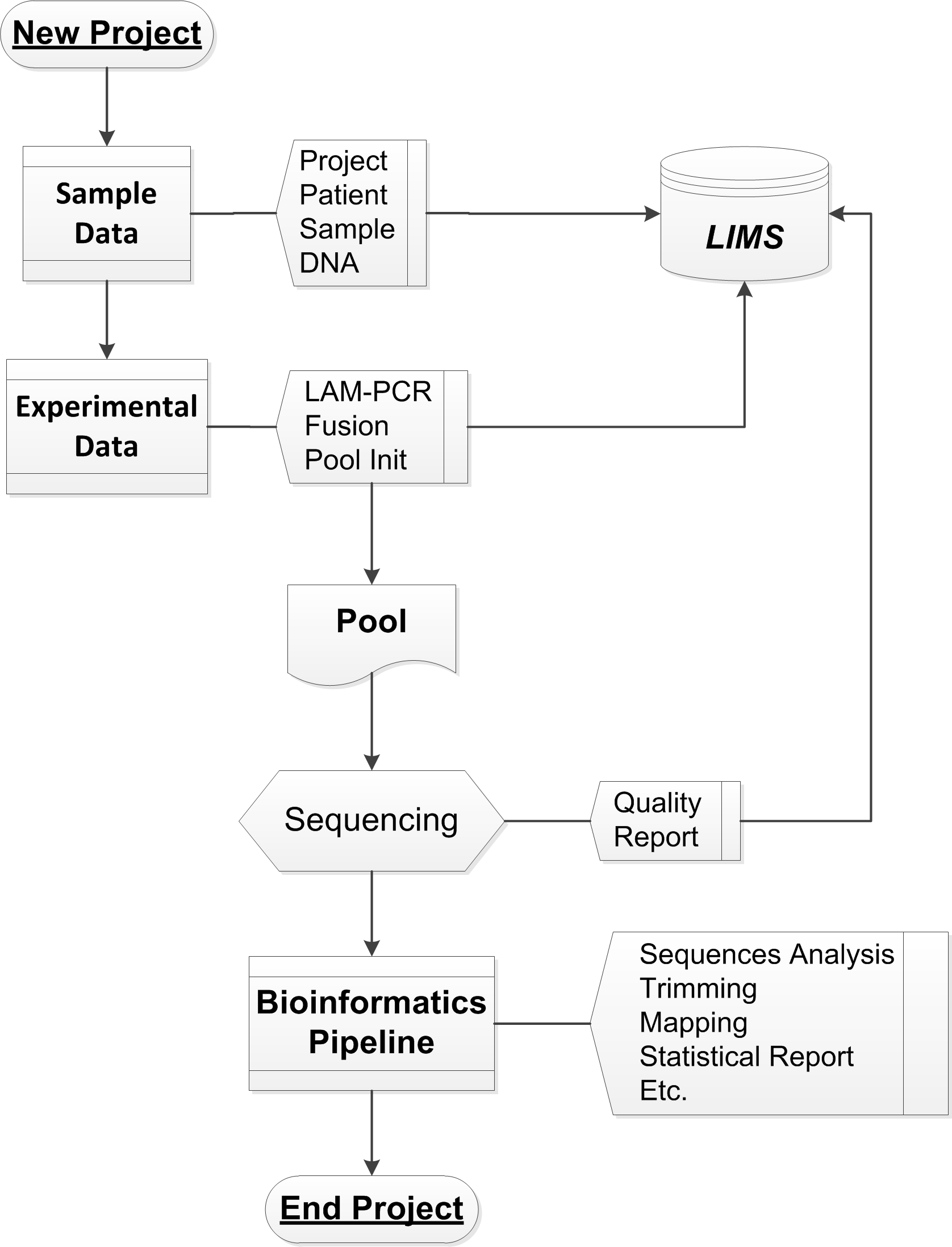

Supplement: Additional file 1 — General data workflow of vector integration sites identification at TIGET Vector Integration Core The standard flow of a vector integration site project in the Vector Integration Core at the Telethon Institute for Gene Therapy in Milan [file 1471-2105-16-S9-S5-S1.docx]
