# Supplementary material for: adLIMS: a customized open source software that allows bridging clinical and basic molecular research studies
Source: BMC Bioinformatics. 2015 Jun 1;16(Suppl 9):S5. doi: 10.1186/1471-2105-16-S9-S5 (PMC4464029; doi:10.1186/1471-2105-16-S9-S5)
Supplement: Additional file 2 — Features' comparison between Bika, LabKey and ADempiere Comparison among the features available in Bika, LabKey and ADempiere. [file 1471-2105-16-S9-S5-S2.docx]

**adLIMS: a customized open source software that allows bridging clinical and basic molecular research studies**

Andrea Calabria^1^, Giulio Spinozzi ^1,2^, Fabrizio Benedicenti^1^, Erika Tenderini^1^, Eugenio Montini^1§^

^1^ San Raffaele Scientific Institute, Division of Regenerative medicine, Stem cells, and Gene therapy - HSR-TIGET - The San Raffaele Telethon Institute for Gene Therapy; Milan, Italy

^2^ Department of Informatics, Systems and Communication (DISCo) - University of Milano-Bicocca (UNIMIB); Milan, Italy

^§^Corresponding author

Email addresses:

AC: [calabria.andrea@hsr.it](mailto:calabria.andrea@hsr.it)

GS: [spinozzi.giulio@hsr.it](mailto:spinozzi.giulio@hsr.it)

FB: [benedicenti.fabrizio@hsr.it](mailto:benedicenti.fabrizio@hsr.it)

ET: [tenderini.erika@hsr.it](mailto:tenderini.erika@hsr.it)

EM: [montini.eugenio@hsr.it](mailto:montini.eugenio@hsr.it)

# Additional files

# Additional file 2

# Features’ comparison between Bika, LabKey and ADempiere.

Comparison among the features available in Bika, LabKey and ADempiere.

| **Sample, inventory, and data management** | **Bika** | **LabKey** | **ADempiere** |
| --- | --- | --- | --- |
| Sample login and management | Y | Y | Y |
| Sample tracking | - | Y | Y |
| Sample and/or result batching | Y | Y | Y |
| Task and event scheduling | - | Y | Y |
| Option for manual result entry | Y | - | Y |
| Multiple data viewing methods | - | Y | Y |
| Data and trend analysis | Y | Y | Y |
| Data and equipment sharing | Y | Y | Y |
| Customizable fields and/or interface | Y | Y | Y |
| Query capability | Y | Y | Y |
| Import data | - | Y | Y |
| Export data to MS Excel | - | Y | Y |
| Data warehouse | - | Y | Y |
| Project and/or task management | - | Y | Y |
| Document creation and/or management | Y | Y | Y |
| Case management | - | Y | Y |
| Workflow management | - | - | Y |
| Specification management | Y | - | Y |
| Customer and supplier management | Y | - | Y |
| **Reporting, barcoding, and printing** | **Bika** | **LabKey** | **ADempiere** |
| Custom reporting | Y | - | Y |
| Report printing | Y | - | Y |
| Label support | Y | - | Y |
| Barcode support | - | - | Y |
| Export to PDF | Y | - | Y |
| Fax integration | Y | - | Y |
| Email integration | Y | Y | Y |
| **Base functionalities** | **Bika** | **LabKey** | **ADempiere** |
| Administrator management | Y | Y | Y |
| Modular | Y | - | Y |
| Alarms and/or alerts | Y | - | Y |
| External monitoring | Y | - | Y |
| Messaging | Y | - | Y |
| Network-capable | Y | - | Y |
| Web client or portal | Y | Y | Y |
| **Graphical User Interface** | **Bika** | **LabKey** | **ADempiere** |
| User Friendly Customization | - | Y | - |
| Widgets | - | - | Y |
| Graphical Themes | - | Y | - |
| **Built-in Analysis** | **Bika** | **LabKey** | **ADempiere** |
| Integration with most common analysis tools | - | Y | - |
| Use personal scripts/program | Y | Y | - |
