# Supplementary material for: adLIMS: a customized open source software that allows bridging clinical and basic molecular research studies
Source: BMC Bioinformatics. 2015 Jun 1;16(Suppl 9):S5. doi: 10.1186/1471-2105-16-S9-S5 (PMC4464029; doi:10.1186/1471-2105-16-S9-S5)
Supplement: Additional file 3 — Customization set-up The report file of all the customization steps required for creating an instance of adLIMS, with an example of table creation and trigger definition. [file 1471-2105-16-S9-S5-S3.docx]

**adLIMS: a customized open source software that allows bridging clinical and basic molecular research studies**

Andrea Calabria^1^, Giulio Spinozzi ^1,2^, Fabrizio Benedicenti^1^, Erika Tenderini^1^, Eugenio Montini^1§^

^1^ San Raffaele Scientific Institute, Division of Regenerative medicine, Stem cells, and Gene therapy - HSR-TIGET - The San Raffaele Telethon Institute for Gene Therapy; Milan, Italy

^2^ Department of Informatics, Systems and Communication (DISCo) - University of Milano-Bicocca (UNIMIB); Milan, Italy

^§^Corresponding author

Email addresses:

AC: [calabria.andrea@hsr.it](mailto:calabria.andrea@hsr.it)

GS: [spinozzi.giulio@hsr.it](mailto:spinozzi.giulio@hsr.it)

FB: [benedicenti.fabrizio@hsr.it](mailto:benedicenti.fabrizio@hsr.it)

ET: [tenderini.erika@hsr.it](mailto:tenderini.erika@hsr.it)

EM: [montini.eugenio@hsr.it](mailto:montini.eugenio@hsr.it)

# Additional files

# Additional file 3

# Customizing ADempiere ERP to *adLIMS*

ADempiere integrates a management system called *Application Data Dictionary* (inherited from the original first project Compiere, http://www.compiere.com/) which allows users to monitor, edit, and add all the constituent elements of the application, from data to interfaces (GUI).

In order to create a custom version of ADempiere (in this case *adLIMS*), we customized ADempiere functionalities through the application dictionary as follows:

1. *Creation of the organization (in our case TIGET) and group (Laboratory):* in ADempiere before to start working with the management system is necessary to make an initial set of corporate information. The role of *system administrator* enables the user to modify global system shared by all companies set up in this system configuration masks, database tables, application dictionary, security parameters and so on. Once a user is logged as administrator, it is enabled to start the process *Initial Client Setup Process* that allows the creation of a new company.
2. *Creation of roles and users (SampleManager, WetManager):* once logged in as organization administrator, create specific role for a user or a group of users in the mask *Role* and add users through the *User* window, compiling all information about them and assign them to the specific group.
3. *Import tables previously generated with SQL:* logged in as system administrator, generate and add in the application dictionary the database schema reported in Additional file 3, or importing all the tables one by one or through the appropriate mask Table and Column in ADempiere naming each table with the specific name in the PostgreSQL DB.
4. *Policy settings of data:* in the same section it is possible to set the permission of data access to the table, thus data, for each user role or user.
5. *Creation of Menu, Window and access settings: Window, Tab & Field:* to create the GUI of our system it is necessary to associate a window or a tab (subsection of a window) for each table. In this way a mask for the data of each table entity is created.
6. *Import of customizations (views and triggers):* the last step is the customization that requires to import in ADempiere views and triggers written procedural language PL/pgSQL. The triggers are automatically loaded in the database but the views must be imported as tables, as previously described.

ADempiere allows the creation of GUI windows through tables exploiting the application dictionary and thus avoiding to write custom code. The application dictionary requires predefined fields in the tables with relative foreign keys:

ad_client_id, ad_org_id, Created, createdby, description, isactive, name, updated, updatedby and value.

Here the SQL code for an exemplificative “project” table creation:

-- Table: adempiere.lims_project

CREATE TABLE adempiere.lims_project

(

ad_client_id numeric(10,0) NOT NULL,

ad_org_id numeric(10,0) NOT NULL,

created timestamp without time zone NOT NULL,

createdby numeric(10,0) NOT NULL DEFAULT 0,

description character varying(255) DEFAULT NULL::character varying,

isactive character(1) NOT NULL DEFAULT 'Y'::bpchar,

name character varying(60) NOT NULL,

updated timestamp without time zone NOT NULL,

updatedby numeric(10,0) NOT NULL,

value character varying(40) NOT NULL,

lims_project_id numeric(10,0) NOT NULL,

lims_vector_id numeric(10,0) DEFAULT NULL::numeric,

lims_specie_id numeric(10,0) DEFAULT NULL::numeric,

starting_date date,

finishing_date date,

CONSTRAINT lims_project_key PRIMARY KEY (lims_project_id),

CONSTRAINT lims_project_isactive_check CHECK (isactive = ANY (ARRAY['Y'::bpchar, 'N'::bpchar]))

)

WITH (

OIDS=FALSE

);

ALTER TABLE adempiere.lims_project

OWNER TO adempiere;

## Creating tuples automatically

As said before a user with *technician* role manages large amount of samples (previously inserted by a user with *SampleManager* role) and for each sample he/she setups a workflow of LAM-PCR experiment. We developed an important feature in *adLIMS* that allows to create and support automatically a LAM-PCR experiment thus avoiding inserting each entry by hand (for each experiment it may be required to insert hundreds of entries). A similar implementation is available to generate the pool for sequencing. We implemented this feature through database triggers.

Here the PL/pgSQL code for trigger related to the LAM-PCR workflow:

CREATE OR REPLACE FUNCTION adempiere.lims_trigger_lam_experiment()

RETURNS TRIGGER AS

$BODY$

DECLARE

my_targetLastID numeric(10,0);

my_adClientLastID numeric(10,0);

my_adOrgLastID numeric(10,0);

my_createdByLast numeric(10,0);

my_updatedByLast numeric(10,0);

my_valueByLast character varying(40);

my_valueByLast_int numeric(10,0);

i numeric(10,0) := 1;

BEGIN

IF(TG_OP='INSERT') THEN

/**

*After insert:

*1) get last ID from target table

*2) check if new.n_lam is not null and is positive int

*3) using new.n_lam create same rows : loop for i=1 to new.n_lam do insert new row with specific IDs

*/

/*1) get last IDs and some values from target table */

SELECT lims_lam_pcr_linear.lims_lam_pcr_linear_id INTO my_targetLastID

FROM adempiere.lims_lam_pcr_linear

ORDER BY lims_lam_pcr_linear.lims_lam_pcr_linear_id DESC

LIMIT 1;

IF (my_targetLastID IS NOT NULL) THEN

SELECT lims_experiment.ad_client_id INTO my_adClientLastID

FROM adempiere.lims_experiment

ORDER BY lims_experiment.ad_client_id DESC

LIMIT 1;

SELECT lims_experiment.ad_org_id INTO my_adOrgLastID

FROM adempiere.lims_experiment

ORDER BY lims_experiment.ad_org_id DESC

LIMIT 1;

SELECT lims_experiment.createdby INTO my_createdByLast

FROM adempiere.lims_experiment

ORDER BY lims_experiment.createdby DESC

LIMIT 1;

SELECT lims_experiment.updatedby INTO my_updatedByLast

FROM adempiere.lims_experiment

ORDER BY lims_experiment.updatedby DESC

LIMIT 1;

SELECT lims_experiment.value INTO my_valueByLast

FROM adempiere.lims_experiment

ORDER BY lims_experiment.value DESC

LIMIT 1;

RAISE NOTICE '>> lims_lam_pcr_linear_id = %', my_targetLastID;

my_valueByLast_int=CAST(my_valueByLast AS numeric);

/*2) check if new.n_lam is not null and is positive int*/

IF (NEW.n_lam>0 AND NEW.n_lam IS NOT NULL) THEN

/*3) using NEW.n_lam create same rows : loop for i=1 to NEW.n_lam do insert NEW row with specific IDs*/

<<loop_n_lam>>

WHILE i <= NEW.n_lam LOOP

-- FOR i in NEW.n_lam LOOP

INSERT INTO adempiere.lims_lam_pcr_linear (ad_client_id, ad_org_id, created, createdby, isactive, name, updated, updatedby, value, lims_lam_pcr_linear_id, lims_project_id, lims_subject_id, lims_dna_id, lims_sample_id, lims_experiment_id, lam_pcr_linear_date) VALUES (my_adClientLastID, my_adOrgLastID, CURRENT_TIMESTAMP, my_createdByLast,'Y', CONCAT(NEW.id_lam_lab,'.',i) , CURRENT_TIMESTAMP, my_updatedByLast, my_valueByLast_int + i, my_targetLastID + i, NEW.lims_project_id, NEW.lims_subject_id, NEW.lims_dna_id, NEW.lims_sample_id, NEW.lims_experiment_id, NEW.experiment_date);

i := i+1;

END LOOP loop_n_lam;

ELSE

RAISE EXCEPTION '[LIMS] ERROR: n_lam = %', NEW."n_lam";

END IF;

RETURN NEW;

END IF;

END IF; /* if TG_OP */

END;

$BODY$

LANGUAGE plpgsql;

CREATE TRIGGER run_lims_experiment

AFTER INSERT

ON adempiere.lims_experiment

FOR EACH ROW

EXECUTE PROCEDURE adempiere.lims_trigger_lam_experiment();
