# Supplementary material for: adLIMS: a customized open source software that allows bridging clinical and basic molecular research studies
Source: BMC Bioinformatics. 2015 Jun 1;16(Suppl 9):S5. doi: 10.1186/1471-2105-16-S9-S5 (PMC4464029; doi:10.1186/1471-2105-16-S9-S5)

**adLIMS: a customized open source software that allows bridging clinical and basic molecular research studies**

Andrea Calabria^1^, Giulio Spinozzi ^1,2^, Fabrizio Benedicenti^1^, Erika Tenderini^1^, Eugenio Montini^1§^

^1^ San Raffaele Scientific Institute, Division of Regenerative medicine, Stem cells, and Gene therapy - HSR-TIGET - The San Raffaele Telethon Institute for Gene Therapy; Milan, Italy

^2^ Department of Informatics, Systems and Communication (DISCo) - University of Milano-Bicocca (UNIMIB); Milan, Italy

^§^Corresponding author

Email addresses:

AC: [calabria.andrea@hsr.it](mailto:calabria.andrea@hsr.it)

GS: [spinozzi.giulio@hsr.it](mailto:spinozzi.giulio@hsr.it)

FB: [benedicenti.fabrizio@hsr.it](mailto:benedicenti.fabrizio@hsr.it)

ET: [tenderini.erika@hsr.it](mailto:tenderini.erika@hsr.it)

EM: [montini.eugenio@hsr.it](mailto:montini.eugenio@hsr.it)

# Additional files

# Additional file 4

# adLIMS Schema (PostgreSQL)

*adLIMS* database schema represented as Entity-Relationship model. Different entities (rectangles) with their associations (lines) are shown. An entity (table) is described by its attributes, e.g. a sample can be specified by its name, date, with primary key/s (PK in bold), foreign key/s (FK in italic) and primary foreign key/s (PF in bold). This schema does not include the ADempiere inner tables, but only the *adLIMS* related, in its connection to the database driven logic. Each entry of the tables is a field stored in our PostgreSQL database.


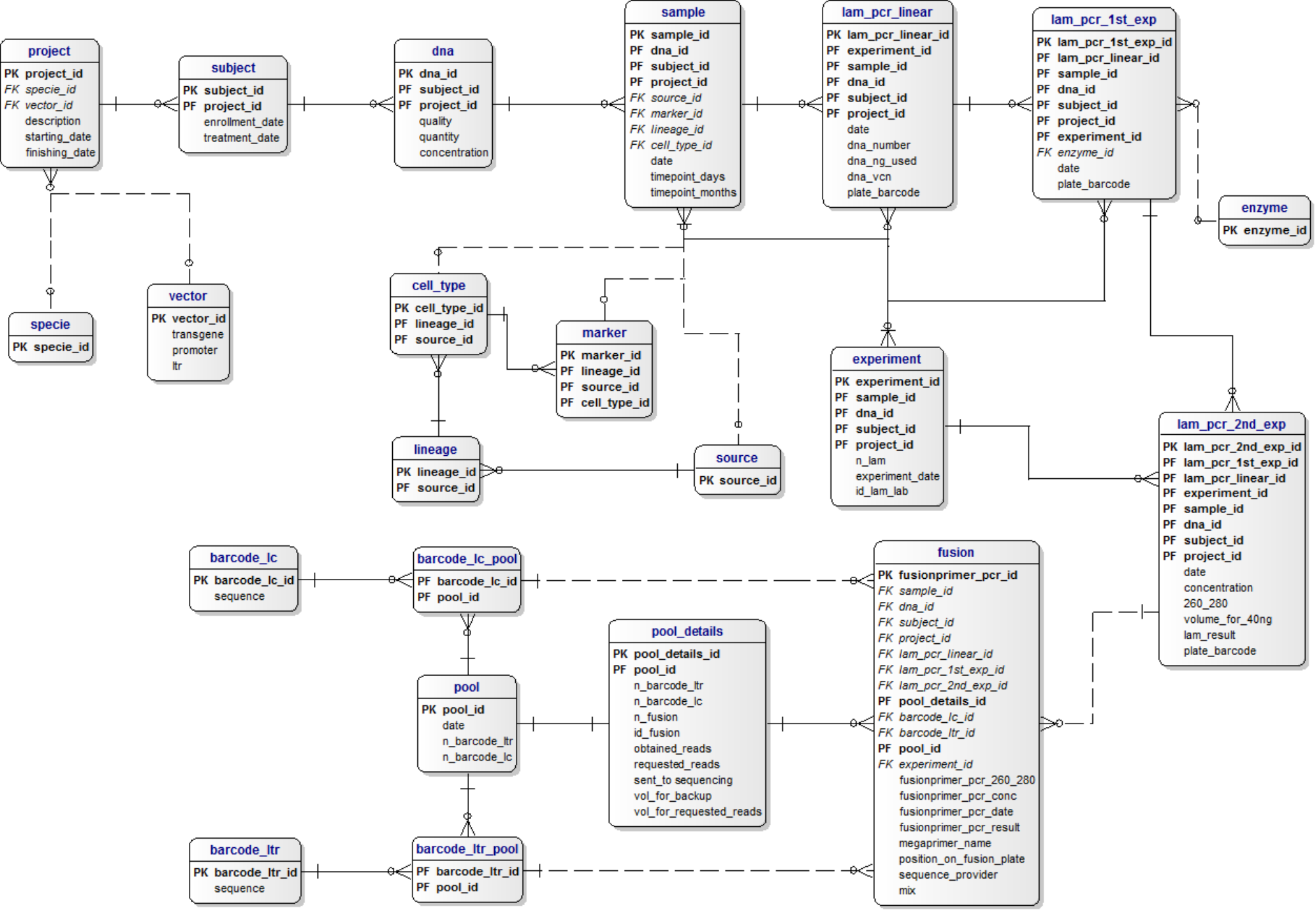

Supplement: Additional file 4 — adLIMS entity-relationship database model adLIMS database schema represented as Entity-Relationship model. [file 1471-2105-16-S9-S5-S4.docx]
