# Supplementary material for: adLIMS: a customized open source software that allows bridging clinical and basic molecular research studies
Source: BMC Bioinformatics. 2015 Jun 1;16(Suppl 9):S5. doi: 10.1186/1471-2105-16-S9-S5 (PMC4464029; doi:10.1186/1471-2105-16-S9-S5)
Supplement: Additional file 5 — Data elements managed by adLIMS In this file we reported all data types inserted in adLIMS, following the workflow in Figure 3. [file 1471-2105-16-S9-S5-S5.docx]

**adLIMS: a customized open source software that allows bridging clinical and basic molecular research studies**

Andrea Calabria^1^, Giulio Spinozzi ^1,2^, Fabrizio Benedicenti^1^, Erika Tenderini^1^, Eugenio Montini^1§^

^1^ San Raffaele Scientific Institute, Division of Regenerative medicine, Stem cells, and Gene therapy - HSR-TIGET - The San Raffaele Telethon Institute for Gene Therapy; Milan, Italy

^2^ Department of Informatics, Systems and Communication (DISCo) - University of Milano-Bicocca (UNIMIB); Milan, Italy

^§^Corresponding author

Email addresses:

AC: [calabria.andrea@hsr.it](mailto:calabria.andrea@hsr.it)

GS: [spinozzi.giulio@hsr.it](mailto:spinozzi.giulio@hsr.it)

FB: [benedicenti.fabrizio@hsr.it](mailto:benedicenti.fabrizio@hsr.it)

ET: [tenderini.erika@hsr.it](mailto:tenderini.erika@hsr.it)

EM: [montini.eugenio@hsr.it](mailto:montini.eugenio@hsr.it)

# Additional files

# Additional file 5

## Data elements accommodated by adLIMS

Following the *adLIMS’* workflow:

**SampleManager Role**:

1. Window - **Cell Forms** (hierarchical):
   1. Tab - ***Source***: Name
   2. Tab - ***Lineage***: Name
   3. Tab - ***Cell Type***: Name
   4. Tab - ***Cell Marker***: Name
2. Window – **Sample** (hierarchical) – **Step 1 on the Workflow**:
   1. Tab – ***Project***: Name, Starting Date, Finishing Date, Vector, Specie.
   2. Tab – ***Subject***: Name, Enrollment Date, Treatment date.
   3. Tab – ***DNA***: Name, Concentration, Quality, Quantity.
   4. Tab – ***Sample***: Name, Date, Timepoint, Cell Marker, Cell Type, Lineage, Source.

**WetManager Role**:

1. Window – **Parameters**:
   1. Tab – ***Specie***: Name
   2. Tab – ***Vector***: Name, LTR, Promoter, Transgene
   3. Tab – ***Barcode LTR***: Name, Sequence LTR
   4. Tab – ***Barcode LC***: Name, Sequence LC
   5. Tab – ***Enzyme***: Name
2. Window – **LAM-PCR** (hierarchical) – **Step 2 on the Workflow**:
   1. Tab – ***Experiment***: Name, N-LAM, LAM ID, Experiment Date, Sample, DNA, Subject, Project
   2. Tab – ***LAM-PCR Linear***: Name, Date, DNA Used, DNA VCN, Plate Barcode.
   3. Tab – ***LAM-PCR 1^st^ Exp***: Name, Date, Plate Barcode
   4. Tab – ***LAM-PCR 2^nd^ Exp***: Name, Date, Concentration, Quality, Volume, LAM Result, Plate Barcode.
3. Window – **Pool** (hierarchical) – **Step 3 on the Workflow**:
   1. Tab – ***Setup***: Name, Date, N-Barcode LTR, N-Barcode LC
   2. Tab – ***Barcode LTR***: Name, Barcode LTR
   3. Tab – ***Barcode LC***: Name, Barcode LC
   4. Tab – ***Pool Details***: Name, N-Barcode-LTR, N-Barcode-LC, N-Fusion, ID-Fusion, Volume.
   5. Tab – ***Fusion***: Name, Quality, Concentration, Date, Result, Position-On-Plate, MIX, Sequence Provider.

**Step 4 on the Workflow:** To proceed to sequencing, it is necessary to have a sample sheet with information about the sample, under what is called NGS sample-sheet. This file can be generated directly from *adLIMS* by a user with *WetManager* role from the table of the sample through the appropriate function for the export.

Note: at every step, in every tab and window, a user can insert any type of attachment, images or binary files. In Figure 3 is shown as an example the loading of an image describing the quality of an aliquot of DNA and the result of a LAM-PCR. Files larger than 2GB cannot be uploaded, so there is no use of FASTQ file, which instead are only stored as an attribute in the appropriate field in the table Pool.
